# Supplementary material for: Coherent optical communications using coherence-cloned Kerr soliton microcombs
Source: Nat Commun. 2022 Feb 28;13:1070. doi: 10.1038/s41467-022-28712-y (PMC8885653; doi:10.1038/s41467-022-28712-y)
Supplement: Supplementary file 1 — Supplementary Information [file 41467_2022_28712_MOESM1_ESM.pdf]

Supplementary Information for "Coherent optical communications using coherence-cloned Kerr soliton microcombs"

Yong Geng[1,3], Heng Zhou[1,3,*], Xinjie Han[1], Wenwen Cui[1], Qiang Zhang[1], Boyuan Liu[1], Guangwei Deng[2], Qiang Zhou[2], and Kun Qiu[1]

[1]Key Lab of Optical Fiber Sensing and Communication Networks, University of Electronic Science and Technology of China, Chengdu 611731, China
[2]Institute of Fundamental and Frontier Sciences, University of Electronic Science and Technology of China, Chengdu 611731, China
[3]These authors contributed equally: Yong Geng, Heng Zhou
[*]Corresponding author: zhouheng@uestc.edu.cn

Supplementary Note 1:
Phase analysis between the transmitter and receiver microcombs

After 50 km fiber transmission, the transmitter comb lines $C_{\mathrm{Tx}}(m)$ when they arrive at the receiver can be expressed as[1,2]:

$$E_0^{\mathrm{Tx}}(t) = \cos[\omega_0(t) \cdot t + \phi_0^{\mathrm{NL}}(t)] \tag{S1}$$

$$E_m^{\mathrm{Tx}}(t) = \cos[(\omega_0(t - T_m) + m \cdot \omega_{\mathrm{rep}}^{\mathrm{Tx}}(t - T_m)) \cdot t + \phi_m^{\mathrm{NL}}(t)] \tag{S2}$$

$$T_m = \frac{k_m \cdot L}{\omega_m} - \frac{k_0 \cdot L}{\omega_0} = \frac{[k_0 + k_0^{'}(m \cdot \omega_{\mathrm{rep}}^{\mathrm{Tx}}) + \frac{k_0^{''}}{2}(m \cdot \omega_{\mathrm{rep}}^{\mathrm{Tx}})^2] \cdot L}{\omega_0 + m \cdot \omega_{\mathrm{rep}}^{\mathrm{Tx}}} - \frac{k_0 \cdot L}{\omega_0} \tag{S3}$$

$E_0^{\mathrm{Tx}}(t)$ is the pump laser field and $E_m^{\mathrm{Tx}}(t)$ is the m-th transmitter comb line. ω_0 denotes the pump laser angular frequency and $\omega_{\mathrm{rep}}^{\mathrm{Tx}}(t)$ the frequency spacing (i.e., soliton repetition rate) of the

transmitter comb $C_{\mathrm{Tx}}(m)$. T_m and ϕ_m^{NL} denote the time delay with respective to the pump field and the nonlinear phase shift experienced by the m-th comb line within the 50 km fiber link.k_0, $k_0^{'}$, $k_0^{''}$ denotes the propagation constant, the group index and second order dispersion of the fiber at the pump laser frequency.

Similarly, the comb lines of the regenerated receiver microcomb C_{Rx} can be expressed as:

$$E_0^{\mathrm{Rx}}(t) = \cos[\omega_0(t - T_{\mathrm{d}}) \cdot t + \phi_0^{\mathrm{NL}}(t)] \tag{S4}$$

$$E_m^{\mathrm{Rx}}(t) = \cos[(\omega_0(t - T_{\mathrm{d}}) + m \cdot \omega_{\mathrm{rep}}^{\mathrm{Rx}}(t)) \cdot t + \phi_0^{\mathrm{NL}}(t)] \tag{S5}$$

Here T_{d} denotes the time delay of the regenerated receiver comb C_{Rx} with respective to the arrived transmitter comb C_{Tx}, caused by the apparatus (e.g., Er^+ fiber, path cord, fiber filters, etc.) within the regeneration setup. Note, that while deriving Supplementary Equation S1-S5 we have assumed that C_{Tx} and C_{Rx} are both in the soliton mode-locking state and therefore all the comb line phases align to the corresponding pump laser phase with negligible phase offsets.

According to Supplementary Equation S1 and S5, the m-th inter-comb beat note frequency between $C_{\mathrm{Rx}}(m)$ and $C_{\mathrm{Tx}}(m)$ can be expressed as:

$$f_m^{\mathrm{beat}}(t) = (\omega_0(t - T_m) + m \cdot \omega_{\mathrm{rep}}^{\mathrm{Tx}}(t - T_m)) - (\omega_0(t - T_{\mathrm{d}}) + m \cdot \omega_{\mathrm{rep}}^{\mathrm{Rx}}(t)) \tag{S6}$$

Under the condition of simultaneous transmission of multiple data channels in a single fiber, the nonlinear impairment experienced by each channel tend to be approximately identical[3], so the nonlinear terms ϕ_m^{NL}, m=0,1,2,3... disappear in Supplementary Equation S6.

Moreover, locking $C_{\mathrm{Rx}}(17)$ to $C_{\mathrm{Tx}}(17)$ via changing the repetition rate $\omega_{\mathrm{rep}}^{\mathrm{Rx}}(t)$ of the receiver microcomb leads to $f_{17}^{\mathrm{beat}}(t) = f_{\mathrm{OPLL}}(t)$, and we have :

$$\omega_{\mathrm{rep}}^{\mathrm{Rx}}(t) = \omega_{\mathrm{rep}}^{\mathrm{Tx}}(t - T_{17}) + \frac{\omega_0(t - T_{17}) - \omega_0(t - T_{\mathrm{d}}) - f_{\mathrm{OPLL}}(t)}{17} \tag{S7}$$

Here $f_{\mathrm{OPLL}}(t)$ denotes the OPLL reference frequency. Then, the m-th inter-comb beat note frequency becomes:

$$\begin{aligned} f_m^{\mathrm{beat}}(t) =& [\omega_0(t-T_m)-\omega_0(t-T_{\mathrm{d}})-\frac{m}{17}(\omega_0(t-T_{17})-\omega_0(t-T_{\mathrm{d}}))] \\ &+ m\cdot[\omega_{\mathrm{rep}}^{\mathrm{Tx}}(t-T_m)-\omega_{\mathrm{rep}}^{\mathrm{Tx}}(t-T_{17})]+\frac{m}{17}f_{\mathrm{OPLL}}(t) \end{aligned} \tag{S8}$$

In particular, the fluctuation of $\omega_{\mathrm{rep}}^{\mathrm{Tx}}$ depends on the pump laser frequency noise, Raman self frequency shift, dispersive-wave induced spectral recoil[4], higher order dispersion, and cavity mode coupling[5]. Detailed discussions of these mechanisms beyond the scope of this manuscript but can be found in prior literature[2,6,7]. Based on prior studies, we can empirically relate the variation of $\omega_{\mathrm{rep}}^{\mathrm{Tx}}$ to the variation of ω_0 using a linear noise transduction ratio $\rho=\mathrm{d}\omega_0/\mathrm{d}\omega_{\mathrm{rep}}^{\mathrm{Tx}}$:

$$\omega_{\mathrm{rep}}^{\mathrm{Tx}}(t)-D_1=\rho\cdot[\omega_0(t)-\omega_{\mathrm{p}}] \tag{S9}$$

Here D_1 is the cavity FSR and ω_{p} the frequency of the pumped cavity mode, both of which are time independent. Using Supplementary Equation S9, $f_m^{\mathrm{beat}}(t)$ becomes:

$$\begin{aligned} f_m^{\mathrm{beat}}(t) =& [\omega_0(t-T_m)-\omega_0(t-T_{\mathrm{d}})-\frac{m}{17}(\omega_0(t-T_{17})-\omega_0(t-T_{\mathrm{d}}))] \\ &+ m\rho\cdot[\omega_0(t-T_m)-\omega_0(t-T_{17})]+\frac{m}{17}f_{\mathrm{OPLL}}(t) \end{aligned} \tag{S10}$$

For the simplest case when the time delay induced by dispersive walk-off T_m and the re-generation apparatus T_{d} are negligible (i.e., $\omega_0(t-T_m)\approx\omega_0(t-T_{\mathrm{d}})\approx\omega_0(t-T_{17})$) , then Supplementary Equation S10 can be simplified to:

$$f_m^{\mathrm{beat}}(t)=\frac{m}{17}f_{\mathrm{OPLL}}(t) \tag{S11}$$

Apparently, Supplementary Equation S11 stands for the fundamental principal of optical frequency division (OFD). More generally, when T_m and T_{d} are taken into consideration, we can write the

phase of the m-th beat note as:

$$\phi_{m^{\text{beat}}}(t) = A \cdot \phi_0(t - T_m) + B \cdot \phi_0(t - T_\text{d}) + C \cdot \phi_0(t - T_{17}) + \frac{m}{17}\phi_\text{OPLL}(t) \tag{S12}$$

$A = (1 + m\rho)$, $B = \frac{m-17}{17}$ and $C = -\frac{m+17m\rho}{17}$. To calculate the linewidth of each inter-comb beat note, its phase variance over a time interval τ needs to be solved[1]:

$$\begin{aligned}
\langle \Delta\phi_m^\text{beat}(t,\tau)^2\rangle =& \langle[\phi_m^\text{beat}(t) - \phi_m^\text{beat}(t-\tau)]^2\rangle \\
&= (A^2 + B^2 + C^2) \cdot \langle \Delta\phi_0(t,\tau)^2\rangle \\
&+ AB\{-2[\langle \Delta\phi_0(t, T_m - T_\text{d})^2\rangle + \langle \Delta\phi_\text{ff}(t, T_m - T_\text{d})^2\rangle] \\
&+ \langle \Delta\phi_0(t, T_m + \tau - T_\text{d})^2 + \langle \Delta\phi_0(t, \tau + T_\text{d} - T_m)^2\rangle\rangle\} \\
&+ BC\{-2[\langle \Delta\phi_0(t, T_\text{d} - T_{17})^2\rangle + \langle \Delta\phi_\text{ff}(t, T_\text{d} - T_{17})^2\rangle] \\
&+ \langle \Delta\phi_0(t, T_\text{d} + \tau - T_{17})^2 + \langle \Delta\phi_0(t, T_{17} + \tau - T_\text{d})^2\rangle\rangle\} \\
&+ AC\{-2[\langle \Delta\phi_0(t, T_m - T_{17})^2\rangle + \langle \Delta\phi_\text{ff}(t, T_m - T_{17})^2\rangle] \\
&+ \langle \Delta\phi_0(t, T_m + \tau - T_{17})^2 + \langle \Delta\phi_0(t, T_{17} + \tau - T_m)^2\rangle\rangle\} \\
&+ (\frac{m}{17})^2\langle \Delta\phi_\text{OPLL}(t,\tau)^2\rangle
\end{aligned} \tag{S13}$$

In the above derivation, we have assumed that the pump laser noise is stationary with ergodicity: $\langle \Delta\phi_0(t,\tau)^2\rangle = \langle \Delta\phi_0(t - T_m,\tau)^2\rangle$. Moreover, since the dispersive walk-off T_m and apparatus delay T_d are both determined by the corresponding fiber length, phase variances due to the random fluctuation of effective fiber lengths are also included as $\langle \Delta\phi_\text{ff}(t,\Delta T)^2\rangle$, $|\Delta T|$ denotes those time difference terms in Supplementary Equation S13. Finally, $\langle \Delta\phi_\text{OPLL}(t,\tau)^2\rangle$ denotes the residual phase variance of the OPLL. Then, the normalized power spectrum of the m-th beat note can be calculated using[8]:

$$S_m(f) = \mathcal{F}\{\exp(\frac{\langle \Delta\phi_0^\text{beat}(\text{t},\tau)^2\rangle}{2})\} \tag{S14}$$

To calculate the results of Supplementary Equation S13 and S14, the statistic properties of the

involved random variables need to be specified. First, we assume that the pump laser phase noise has a Gaussian probability density function[8]:

$$f(\Delta\phi_0) = \frac{1}{2\pi\sqrt{\Delta f\tau}} \cdot e^{-\frac{\Delta\phi_0^2}{4\pi\Delta \mathrm{f}\tau}} \tag{S15}$$

Δf is the Lorentz linewidth of the laser. According to Supplementary Equation S15 the phase evolution of the pump laser has a zero mean value and a variance of:

$$\langle\Delta\phi_0(t,\tau)^2\rangle = 2\pi\Delta f\tau \tag{S16}$$

Second, we assume that the random phase variation $\langle\Delta\phi_{\mathrm{ff}}(t,\Delta T)^2\rangle$ induced by fiber length fluctuation also has a white Gaussian distribution with zero mean value and a time-dependent variance of:

$$\langle\Delta\phi_{ff}(t,\Delta T)^2\rangle = (\omega_0|\Delta T|\nu)^2\tau \tag{S17}$$

Here we set $\nu = 1 \times 10^{-10}\mathrm{s}^{-1}$, implying that, over a time interval of 1 second, the variance of the fiber length fluctuation is 1×10^{-20} with respect to its original length. Such small phase errors caused by fiber fluctuation are mainly responsible for the low-frequency noise (<±3.0 kHz) in the inter-comb beat note spectra, which, however, has no essential influence to the high-speed coherent data receiving. And third, the OPLL residual phase error $\langle\Delta\phi_{\mathrm{OPLL}}(t,\tau)^2\rangle$ is set in the power spectral domain as a white noise floor at -60 dB (normalized to the beat note power), based on our experimental measurement of the locked 17th inter-comb beat note spectrum, as shown in main text Fig. 1c.

Supplementary Figure 1a shows the calculated $S_m(f)$ using Supplementary Equation S13 to S17 and our experimental parameters (see Supplementary Figure 1 caption). It is confirmed that two-point locking indeed leads to substantial suppression of the inter-comb beat note linewidth comparing with the case without two-point locking (corresponding to the case with $A = 1 + m\rho$, $B = -1 + m\rho$ and $C = 0$), agreeing well with the experimental measurements. The main noise

source originates from the OPLL, which evolves as the function of $(\frac{m}{17})^2$, following the law of OFD. Moreover, for a 1 kHz linewidth pump laser as used in our experiment, the dispersive walk-off causes no sizable linewidth broadening to the inter-comb beat notes even if the fiber length is increased to 5000 km (see Supplementary Figure 1b). The influence of dispersive walk-off only becomes obvious when the fiber length is set to longer than 5000 km and simultaneously the pump laser linewidth is set to bigger than 1 MHz (see Supplementary Figure 1c). To that extent, we speculate that dispersive walk-off within the fiber link is not a major limitation to the two-point locking scheme, as long as the transmission distance is not too long (i.e., <1000 km) and the pump laser has sufficiently low noise. For ultra-long haul transmission (e.g., >10000 km), a lot more issues need to be reconsidered, including the fidelity of two-point locking, sophisticated dispersion management, and other link impairments compensation, which will be investigated in our follow-up studies.

Finally, it is worth mentioning that the mechanism for the regeneration of soliton microcombs is fundamentally different from the parametric comb regeneration reported in[1]. For the scheme of parametric comb regeneration, fiber dispersion induces phase offset between the two pilot tones, and such phase offset is inevitably multiplexed by m times as the m-th parametric comb line is generated, causing considerable broadening of the comb linewidth when m becomes large. In comparison, for the regeneration of soliton mode-locked microcomb C_{Rx}, the linewidth of each comb line is not influenced by the fiber dispersive walk-off (see Supplementary Equation S5), since C_{Rx} is solely generated by the conveyed monochromatic pump laser. In addition, as can be seen from Supplementary Equation S8, dispersive walk-off gets involved during the process of locking $C_{\mathrm{Rx}}(17)$ to the pilot tone $C_{\mathrm{Tx}}(17)$. Particularly, when the $C_{\mathrm{Rx}}(17)$ is locked to the pilot tone $C_{\mathrm{Tx}}(17)$, the other comb lines $C_{\mathrm{Rx}}(17)$, m=1,2,3,4... are not simultaneously locked to the corresponding $C_{\mathrm{Tx}}(m)$, but subjected to multiple time delays (i.e., T_m, m=1,2,3,4...) due to dispersive walk-off. The overall influence of these time delay terms to the inter-comb beat note linewidth is determined by Supplementary Equation S13, as discussed above.

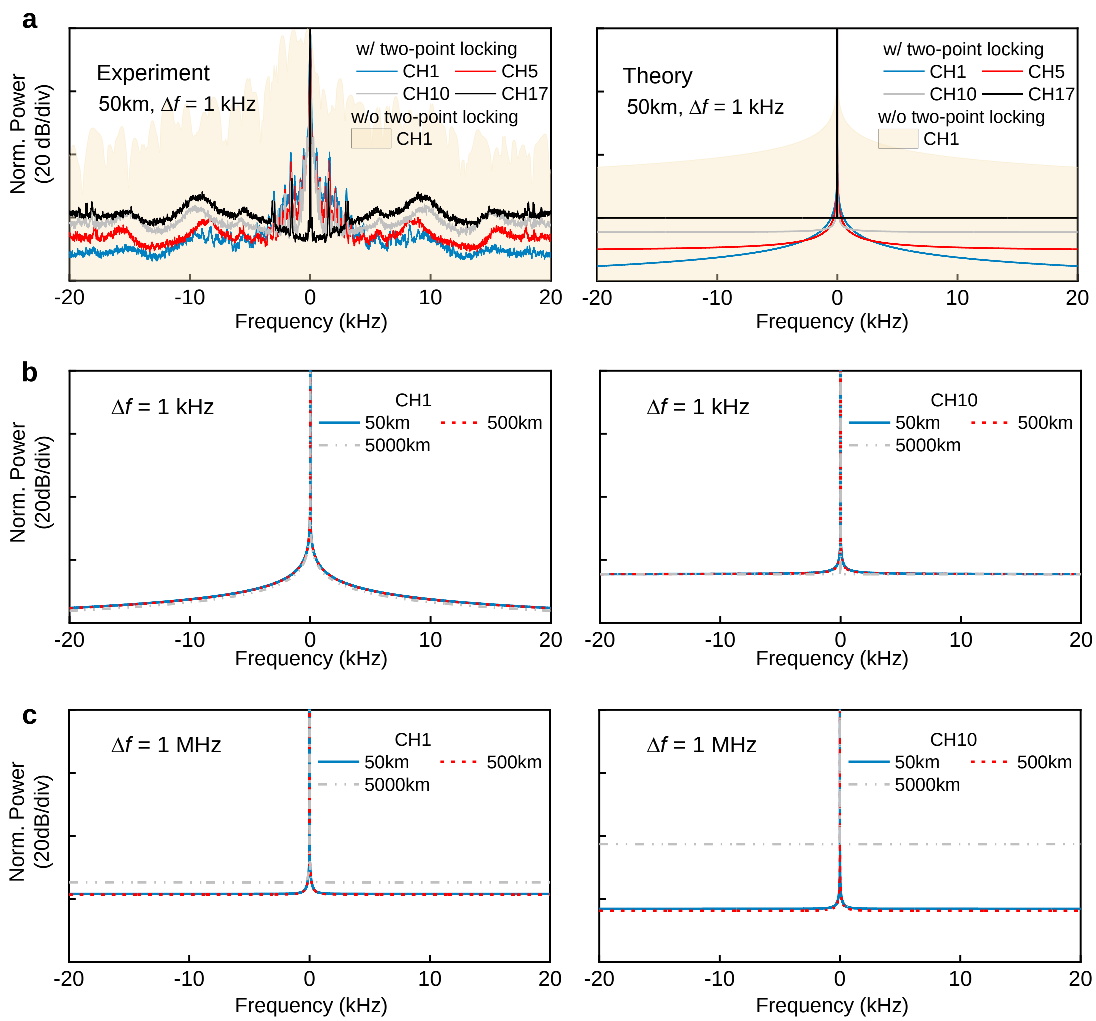

Supplementary Figure 1: **Theoretical phase analysis between the transmitter and receiver microcombs. a.** Comparison of experimental and theoretical inter-comb beat note spectra. **b.** Theoretical inter-comb beat note spectra calculated with 1 kHz pump linewidth. **c.** Theoretical inter-comb beat note spectra calculated with 1 MHz pump linewidth. Parameters used in the theoretical calculation are: ω_0= 193.4 THz, $k_0 = 6.0\times10^6 m^{-1}$, $k_0^{'} = 5.0\times10^{-9}\mathrm{s}\cdot\mathrm{m}^{-1}$, $k_0^{''} = 2.5\times10^{-26}\mathrm{s}^2\cdot\mathrm{m}^{-1}$, $T_\mathrm{d} = 500$ ns, $\rho = 0.02$.

Supplementary Note 2:
Influence of fiber nonlinearity on microcomb re-generation and data interconnect

As mentioned in the main text, the pump laser $C_{\mathrm{Tx}}(0)$ and pilot tone $C_{\mathrm{Tx}}(17)$ of the transmitter comb propagate through the 50 km fiber link together with the high speed data signals. A matter of concern is that the data signals may impose linewidth broadening to them via cross-phase modulation (XPM) and may degrade their spectral purity as the conveyed pump laser and reference pilot[1]. Here we conduct numerical simulation to investigate how $C_{\mathrm{Tx}}(0)$ and $C_{\mathrm{Tx}}(17)$ are impacted by fiber nonlinearity,using the conventional nonlinear Schrödinger equation (NLSE) that models data signal transmission in optical fiber[9]

$$\frac{\partial E}{\partial z} = -\frac{1}{2}\alpha E - i\frac{\beta_2}{2}\frac{\partial^2 E}{\partial T^2} + \frac{\beta_3}{3}\frac{\partial^3 E}{\partial T^3} + i\gamma|E|^2 E + \frac{\gamma}{\omega_0}\frac{\partial}{\partial T}|E|^2 E \tag{S18}$$

Herein $\beta_2 = -20 \times 10^{-27}$ $\mathrm{s^2/m}$ and $\beta_3 = 1.5 \times 10^{-40}$ $\mathrm{s^3/m}$ is the second-and third-order fiber dispersion respectively; $\alpha = 0.2$ dB/km is the loss coefficient, $\gamma = 1.0\ \mathrm{W^{-1} \cdot km^{-1}}$ is the nonlinear coefficient of fiber; the last term stands for self-steepening effect; Raman effect is excluded considering that the relevant laser wavelengths are all within the C-band. The NLSE is numerically solved via the split-step Fourier method using commercial simulation software kit (Optisystem). The simulation contains 20 channels with 100 GHz channel spacing, and each channel is of 21 GBaud single-polarization 16-QAM signal. The 0th and 17th frequency bins are not modulated to mimic the pump laser $C_{\mathrm{Tx}}(0)$ and the pilot tone $C_{\mathrm{Tx}}(17)$. The data on each channel are uncorrelated among the other channels. The power of each data channel is set to 0 dBm, in consistency with the experimental situation. The fiber length is 50 km in our experiment without intermediate amplification.

Supplementary Figure 2 shows the simulation results. It is seen that when the fiber chromatic dispersion is turned off in the simulation, XPM induces sizable temporal phase drifts on the pump laser and pilot tone. In our simulation the temporal phase drifts are extracted by beating them with a zero-linewidth laser tone thus converting their phase fluctuations into amplitude fluctuations. To the contrary, when the fiber chromatic dispersion is turned on, XPM induced phase fluctuations become much smaller. As interpreted in the main text, chromatic dispersion of the fiber induces spatiotemporal walk-off among signals at different wavelengths (i.e., different data channels) along

the transmission link, therefore, XPM imposed to the pump laser $C_{\mathrm{Tx}}(0)$ and pilot tone $C_{\mathrm{Tx}}(17)$ from different data channels are smoothed out as a quasi-constant phase envelop without high frequency component, which would not impact their efficacy as pump laser and pilot tone.

To confirm the above numerical analysis, we conduct a separate experiment where the 20 data channels are transmitted to the receiver within a different fiber from the pump laser $C_{\mathrm{Tx}}(0)$ and pilot tone $C_{\mathrm{Tx}}(17)$, so that to totally eliminate the XPM interactions between data channels and the pump/pilot tones. As shown in Supplementary Figure 2c, data receiving signal-to-noise ratio (SNR) and bit-error ratio (BER) are identical regardless of whether one fiber or two fibers are adopted, confirming that XPM causes no prominent influence in our system.

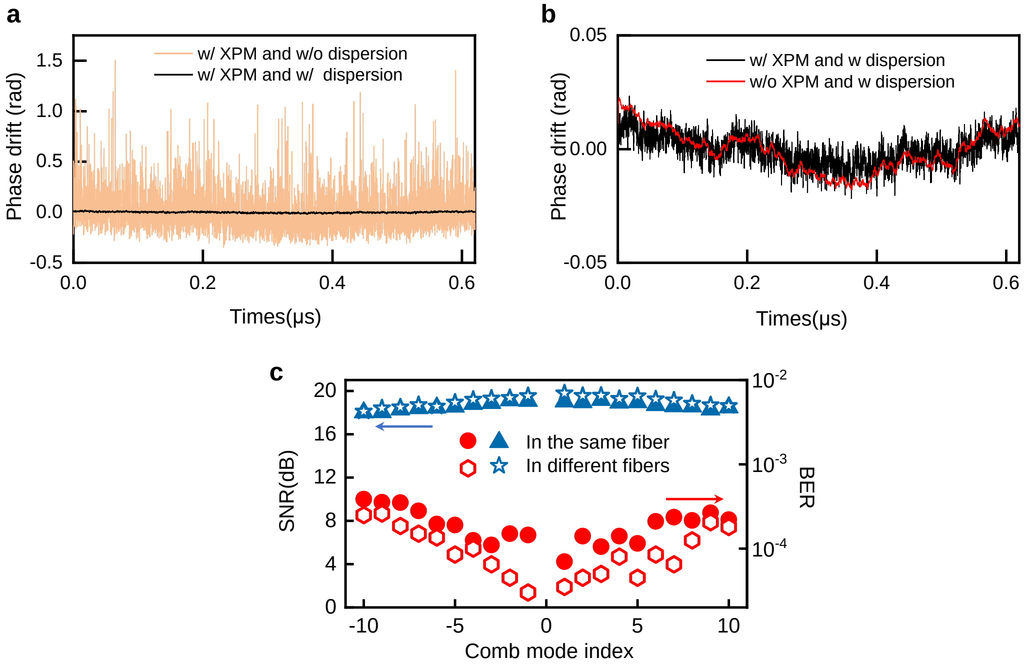

Supplementary Figure 2: **Simulation results of phase degradation induced by fiber nonlinearity. a.** Comparison of the phase deterioration by fiber XPM effect between the models including and excluding the XPM effect. **b.** Comparison of phase deterioration between the models including and excluding the XPM effect. **c.** Comparison of experimental measured data receiving SNR and BER when 20 data channels and the pump/pilot tones are transmitted in the same fiber or in different fibers.

Supplementary Note 3: Quantitative analysis of FOE and CPE simplification enabled by coherence-cloned microcombs

In this section, we conduct quantitative analysis about the saving of digital operations regarding FOE and CPE realized by using coherence-cloned microcombs as carriers and LOs.

First, when independent lasers are used as carrier and LO, FOE algorithm is a necessity to trace the local oscillator frequency offset (LOFO). Supplementary Figure 3d summarizes the digital operations needed for conventional FOE algorithm based on the typical fourth-power fast-Fourier transformation (4-th FFT)[10,11], therein N_{FOE} is the number of symbols used for FFT, and R is the data baud rate. The flow-chart of the 4-th FFT algorithm is plotted in Supplementary Figure 3a. For example, setting the target resolution as 10 MHz and R =21 Gbaud, the FOE requires N_{FOE}=525 symbols, 14740 real multiplications, 16857 real additions, and 525 comparisons for a single data channel. In comparison, it has been demonstrated in the main text that coherence-cloned microcombs have sub-kHz mutual frequency stability (much higher than the precision of conventional FOE, see Fig.2f and 2g in the main text), therefore, the FOE process for tracking the LOFO can be totally dispensed with, and all the digital operations regarding FOE can be saved (i.e., 0 multiplications, 0 additions, and 0 comparisons) for all the 20 channels adopted in our experiment.

Second, regarding CPE, the algorithm flow charts and the digital operations needed for typical CPE algorithms (i.e., pilot-aided CPE and BPS: blind phase search) are presented in Supplementary Figure 3[11,12]. For example, if BPS is used with block size N_{BPS}=32 and tested phases B=32, each CPE process requires 6272 real multiplications, 6178 real additions, 32 comparisons and 1056 decisions. As shown in Supplementary Figure 4a below, for independent laser carrier and LO, CPE needs to be updated each every 10 data blocks to satisfy the BER threshold of 3.8e-3, while for coherence-cloned microcombs, CPE rate can be reduced to each every 12500 blocks for channel 1 to channel 5, implying more than 1000× reduction of the DSP operations comparing with independent carrier and LO lasers. As for pilot aided CPE, the operation number for each CPE process is smaller (see Supplementary Figure 3d), so the overall saving of digital operations are fewer for microcombs. However, the much lower pilot-aided CPE rate also means that much fewer pilot symbols are sent, which in turn brings about 1000× reduction of the pilot overhead.

Besides, it is worthy mentioning that, for our 50 km interconnect experiment, only static equalizers were used to retrieve the single polarization 16-QAM data, since the change of link impairments were considered slow in our laboratory environment. Nevertheless, practical coherent receivers usually use adaptive equalizer modules to compensate rapidly-changing link impairments, which could possibly be able to simultaneously compensate the small residual phase variations between the coherence-cloned transmitter and receiver microcombs. As shown in Supplementary Figure 4c, when decision-directed least-mean-square (DD-LMS) adaptive equalizer is implemented to retrieve the 16-QAM data collected in our experiment (parameters of DD-LMS is given in Supplementary Figure 4c caption), the date receiving BER can be controlled well below the desired threshold from CH 1 to CH 10, without conducting CPE for any of the channels. However, when coherence-cloned microcombs are adopted in a realistic network, either the adaptive equalizer should replace or work collaboratively with the CPE module depends on the specific system parameters (such as the fiber link length, channel impairments, polarization sensitivity, cost and power budget, etc.), which we believe is an important topic to follow up.

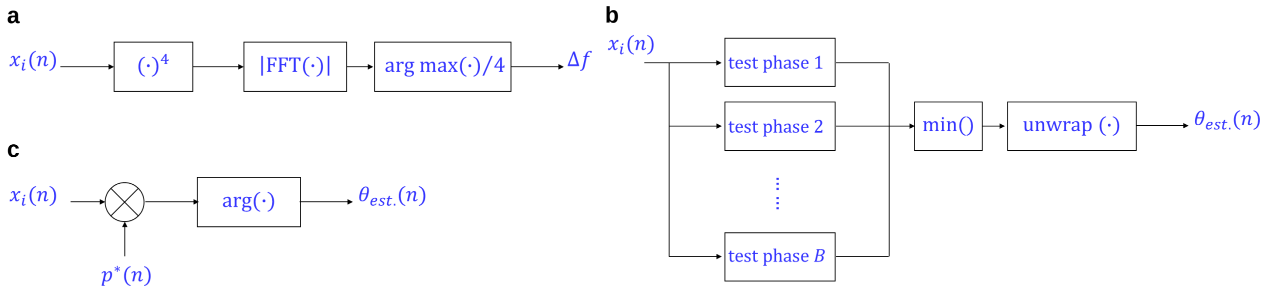

d **Digital operations required by typical FOE and CPE algorithms**

	**Real Multiplications**	**Real Additions**	**Comparisons**
**FOE (4-th FFT)**	$(2N_{\mathrm{FOE}} log_2(N_{\mathrm{FOE}}) + 10N_{\mathrm{FOE}} + 2)$	$(3N_{\mathrm{FOE}} log_2(N_{\mathrm{FOE}}) + 5N_{\mathrm{FOE}})$	N_{FOE}
**BPS-based CPE**	$6BN_{\mathrm{BPS}} + 4N_{\mathrm{BPS}}$	$6BN_{\mathrm{BPS}} - B + 2 + 2N_{\mathrm{BPS}}$	B
**Pilot-aided CPE**	$8N_{\mathrm{pilot}}$	$4N_{\mathrm{pilot}}$	N_{pilot}

Supplementary Figure 3: Flow charts and required digital operations for typical FOE and CPE algorithms. a. Flow chart for 4-th FFT FOE algorithm. **b.** Flow chart for pilot-based CPE algorithm. **c.** Flow chart for BPS based CPE algorithm. **d.** Digital operations required by different FOE and CPE algorithms.

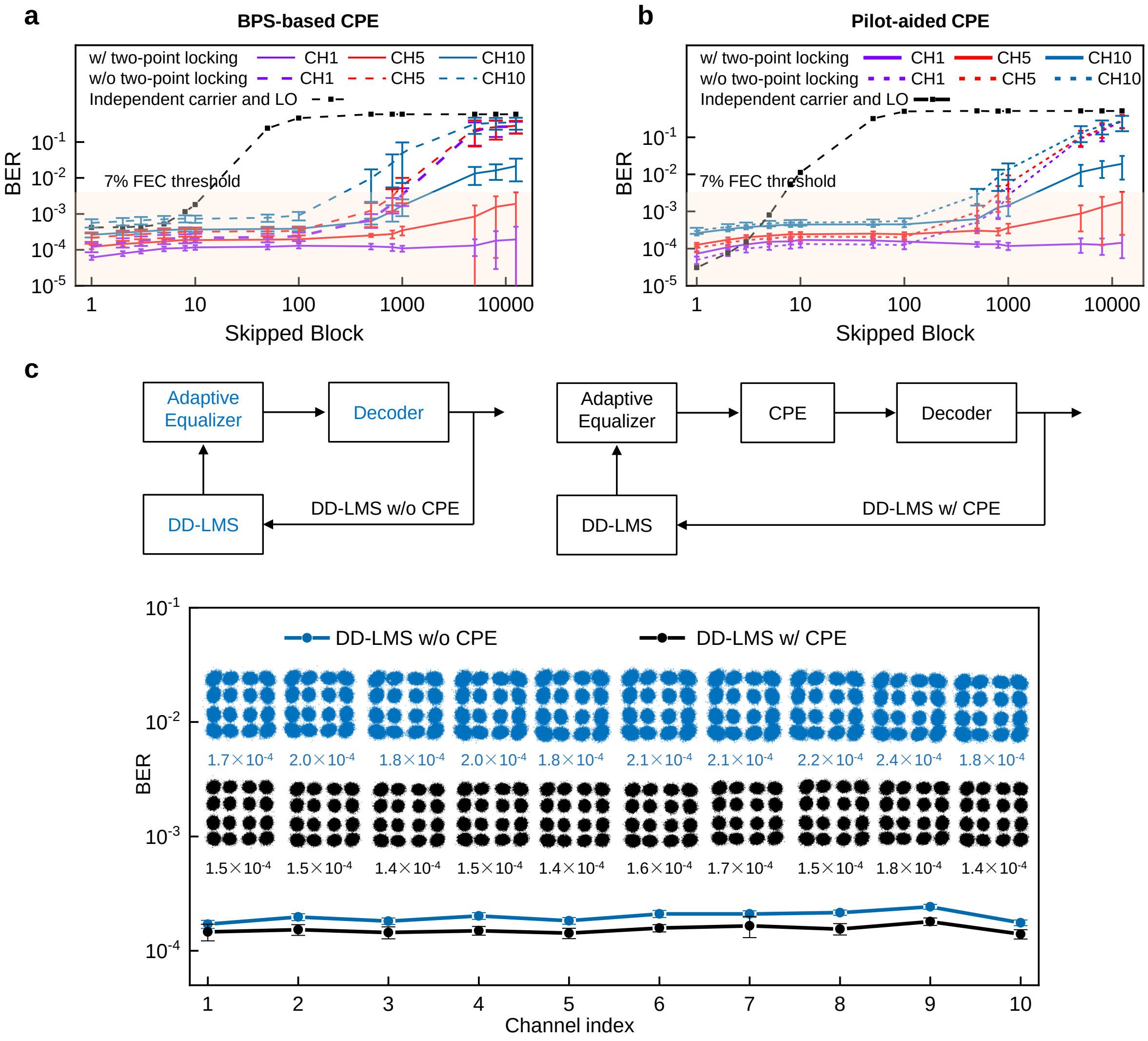

Supplementary Figure 4: **Coherent data performance of different CPE algorithms. a.** Measured BER as a function of BPS-based CPE rate. **b.** Measured BER as a function of pilot-aided CPE rate. **c.** Measured BER using decision-directed adaptive equalizer with or without CPE in the loop. The DD-LMS equalizer contains 17 taps and the step size is 0.0005. The taps are pre-converged using 500 training symbols, and then the equalizer is switched to the decision-directed mode to trace the slow phase drift between C_{Tx} and C_{Rx}.

Supplementary Note 4:
Optical phase lock loop for two-point locking of the transmitter and receiver microcombs

The schematic of the optical phase lock loop (OPLL) for locking $C_{\mathrm{Tx}}(17)$ and $C_{\mathrm{Rx}}(17)$ is presented in Supplementary Figure 5. The ~941.1 MHz beat note between the $C_{\mathrm{Tx}}(17)$ and $C_{\mathrm{Rx}}(17)$ is produced using a fast photodetector, which is then down mixed within a double-balanced mixer to zero with a 941.101 MHz local oscillator signal from a signal synthesizer. The down mixed signal is low-pass filtered and used as the error signal in a proportional–integral–derivative (PID) controller (Newport LB1005), which generates the control signal to actuate the amplitude modulation of the auxiliary laser for generating and tuning C_{Rx}, with a feedback loop bandwidth of 100 kHz. The actuation response speed of the repetition rate of C_{Rx} is considered faster than the loop bandwidth, as instantaneous change of the pump laser detuning can be imposed by the change of intra-cavity power of the auxiliary laser through the effect of XPM[13]. The residual phase noises from the OPLL and the signal synthesizer are considered responsible for the imperfect phase coherence between the carrier and LO (see Fig.1 and Fig.3 of the main text) after two-point locking.

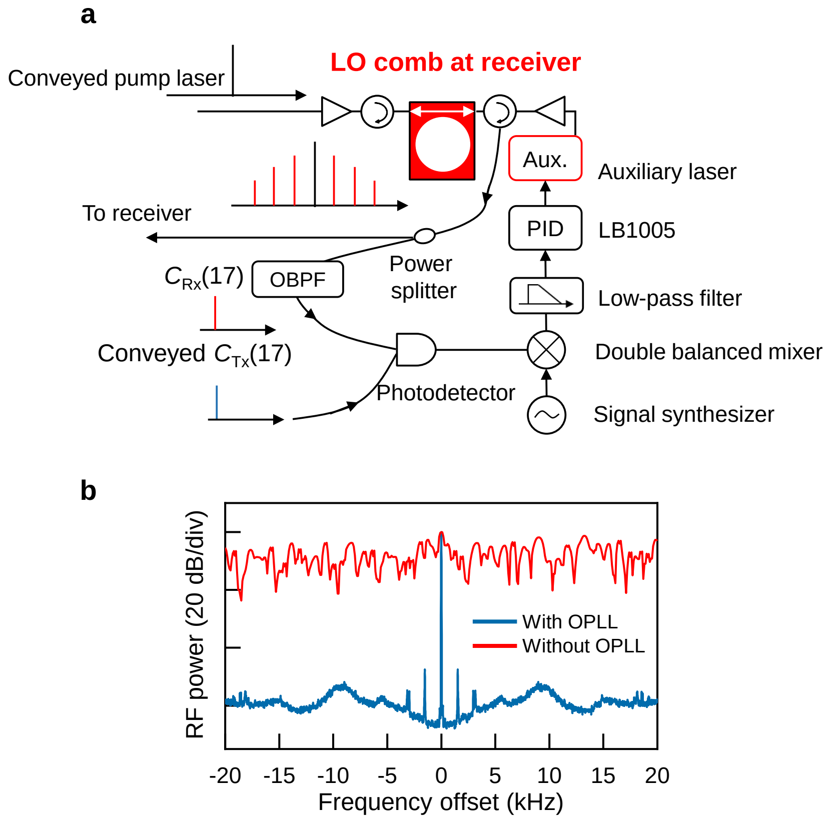

Supplementary Figure 5: **a.** Experimental setup of the OPLL for locking of $C_{\mathrm{Tx}}(17)$ and $C_{\mathrm{Rx}}(17)$. **b.** Comparison of the beat notes between $C_{\mathrm{Tx}}(17)$ and $C_{\mathrm{Rx}}(17)$ with and without using OPLL.

Supplementary References

[1] Lorences-Riesgo, A., Eriksson, T. A., Fülöp, A., Andrekson, P. A. & Karlsson, M. Frequency-comb regeneration for self-homodyne superchannels. *J. Lightwave Technol.* **34**, 1800-1806 (2016).

[2] Lei, F., Ye, Z., Fülöp, A. & Torres-Company, V. Fundamental optical linewidth of soliton microcombs. preprint at https://arxiv.org/abs/2102.05517 (2021).

[3] Lundberg, L. et al. Phase-coherent lightwave communications with frequency combs. *Nat. Commun.* **11**, 201 (2020).

[4] Yi, X. et al. Single-mode dispersive waves and soliton microcomb dynamics. *Nat. Commun.* **8**, 14869-14869 (2017).

[5] Matsko, A. B. & Maleki, L. Noise conversion in Kerr comb RF photonic oscillators. *J. Opt. Soc. Am. B* **32**, 232-240 (2015).

[6] Bao, C. et al. Soliton repetition rate in a silicon-nitride microresonator. *Opt. Lett.* **42**, 759-762 (2017).

[7] Drake, T. E., Stone, J. R., Briles, T. C. & Papp, S. B. Thermal decoherence and laser cooling of Kerr microresonator solitons. *Nat. Photon.* **14**, 480-485 (2020).

[8] Kikuchi, K. Characterization of semiconductor-laser phase noise and estimation of bit-error rate performance with low-speed offline digital coherent receivers. *Opt. Expres* **20**, 5291-5302 (2012).

[9] Agrawal, G. P. *Nonlinear Fiber Optics*, 5th edn (Academic Press, Boston, 2013).

[10] Yang, T. et al. Hardware-efficient multi-format frequency offset estimation for M-QAM coherent optical receivers. *IEEE Photonics Technol. Lett.* **30**, 1605-1608 (2018).

[11] Faruk, M. S. & Savory, S. J. Digital signal processing for coherent transceivers employing multilevel formats. *J. Lightwave Technol.* **35**, 1125-1141 (2017).

[12] Ke, J. H. et al. Linewidth-tolerant and low-complexity two-stage carrier phase estimation for dual-polarization 16-QAM coherent optical fiber communications. *J. Lightwave Technol.* **30**, 3987-3992 (2012).

[13] Ghalanos, G. N. et al. Kerr-nonlinearity-induced mode-splitting in optical microresonators. *Phys. Rev. Lett.* **124**, 223901 (2020).
